# Cell biology of *Candida albicans*–host interactions

**DOI:** 10.1016/j.mib.2016.08.006

**Published:** 2016-12

**Authors:** Alessandra da Silva Dantas, Kathy K Lee, Ingrida Raziunaite, Katja Schaefer, Jeanette Wagener, Bhawna Yadav, Neil AR Gow

**Affiliations:** The Aberdeen Fungal Group, School of Medicine, Medical Science and Nutrition, Institute of Medical Sciences, University of Aberdeen, Aberdeen AB252ZD, UK

## Abstract

*Candida albicans* is a commensal coloniser of most people and a pathogen of the immunocompromised or patients in which barriers that prevent dissemination have been disrupted. Both the commensal and pathogenic states involve regulation and adaptation to the host microenvironment. The pathogenic potential can be downregulated to sustain commensalism or upregulated to damage host tissue and avoid and subvert immune surveillance. In either case it seems as though the cell biology of this fungus has evolved to enable the establishment of different types of relationships with the human host. Here we summarise latest advances in the analysis of mechanisms that enable *C. albicans* to occupy different body sites whilst avoiding being eliminated by the sentinel activities of the human immune system.

## Highlights

• The cell biology of *Candida albicans* is adapted both for life as a commensal and as a pathogen.
• *C. albicans* can either downregulate or upregulate virulence properties in the human host.
• This fungus modulates the activity of phagocytes to enable its own survival.
• *Candida* is metabolically flexible enabling it to survive in multiple niches in the host.

**Current Opinion in Microbiology** 2016, **34**:111–118

This review comes from a themed issue on **Parasitic and fungal diseases**

Edited by **Gero Steinberg**

For a complete overview see the Issue and the Editorial

Available online 28th September 2016

**http://dx.doi.org/10.1016/j.mib.2016.08.006**

1369-5274/© 2016 The Authors. Published by Elsevier Ltd. This is an open access article under the CC BY-NC-ND license (http://creativecommons.org/licenses/by-nc-nd/4.0/).

## Introduction

*Candida albicans* is the commonest serious fungal pathogen of humans, variously reported as causing between 250 000 and 400 000 deaths per annum worldwide as well as extensive morbidity of around 100 million episodes of recurrent vaginitis [1•, 2]. This fungus is a classical opportunistic pathogen residing harmlessly as a commensal in approximately 50% of individuals [1^•^], kept in check by our immune system and a protective bacterial microbiome of the gut and other mucosal surfaces [2, 3, 4]. In this review we survey latest advances in the cell biology of *C. albicans* that underpins its ecology as an organism exploring the interface between commensalism and pathogenesis.

## Life as a successful saprophyte

*C. albicans* is a largely asexual fungus, but never-the-less it is morphologically and physiologically a highly variable and adaptable fungus. It is pleomorphic — being able to grow either as a budding yeast, or as a pseudomycelium of elongated and conjoined yeast cells or as true hyphae formed of generate parallel-sided tip-growing filaments [5]. It also exhibits a non-sexual form of variation called phenotypic switching that can generate stable cell and colony variants with distinct properties [6]. *C. albicans* can thrive in different host niches (gut, vagina, oral mucosa, skin) without causing disease. This observation suggests it is adapted for commensalism. GI tract colonization seems to involve predominantly carriage of the yeast form of the fungus, and low level systemic dissemination in the gut can occur even in yeast-locked mutants [7]. However, a mouse model of stable gastrointestinal candidiasis demonstrated that passage of *C. albicans* through the mammalian gut leads to the transition to a modified yeast cell, ‘GUT phenotype’ (gastrointestinally induced transition), that expresses a specialised transcriptome in the digestive tract to promote assimilation of common nutrients in the bowel. These GUT cells are morphologically altered and suppress the propensity for tissue invasion and the expression of certain virulence traits [8^••^]. Therefore, the commensal state does not solely depend on host immune status and is also supported by organism-specific adaptations mediated by transcriptional changes in the host-associated commensal state [9].

Changes in the availability of nutrients in the gut due to dietary intake, can further impact the abundance of *Candida* [10], and changes in diet or gut fungal microbiota may lead to dysbiosis and inflammatory pathologies such as Crohn's disease [11, 12]. Although *Candida* colonisation may promote gut inflammation under certain circumstances, chitin, a component of the *Candida* cell wall, has anti-inflammatory properties and has the potential to ameliorate inflammatory bowel disease (IBD) if exposed in the gut [13]. Also host mutations in, for example, fungal immune recognition receptors such as dectin-1, the immune signalling molecule CARD9 and the inflammatory cytokine IL-22 result in susceptibility to colitis and other forms of IBD [14, 15]. In a mouse model, tryptophan metabolites from some specific members of the gut microbiome were shown to be able to suppress *C. albicans* colonisation by controlling secretion of IL-22 in stomach [16]. Probiotic supplements are also known to suppress the fungal mycobiome [17]. Therefore the commensal status of *Candida* is also related to the host microbiome as a whole and the immune status of the host.

Collectively, these observations suggest that gut commensalism of *Candida* is related to both intrinsic factors (fungal gene regulation, cell morphology, adaptation, fungal burden) and extrinsic factors (competitive microbiome, diet, host immune status). We know less about the factors affecting colonisation at other body sites although it is likely that a similar complex array of factors sustain the commensal state in these host niches.

## Life as a successful pathogen

During the course of infection *C. albicans* colonizes various host niches, with differences, for example, in nutrient availability, pH, hypoxia and CO_2_ levels [18, 19]. One of the key features establishing *C. albicans* as a successful pathogen is its adaptability to successfully thrive in these different conditions [18]. Host microenvironments have heterogeneous carbon sources and, as it traverses through different host niches, *C. albicans* can adapt to use alternative carbon sources simultaneously, for its survival and virulence [18, 19, 20]. This adaptation has resulted from the absence of catabolite inactivation due to the rewiring of ubiquitination sites in metabolic enzymes [21•, 22]. The metabolic flexibility of *Candida* also contributes to the alterations in the cellular proteome and secretome [23], and its ability to undergo yeast to hyphal transition, white-opaque switching [24], biofilm formation, as well as its adhesion characteristics [23, 25], and the capacity for cell wall remodelling [19, 20, 23, 26]. Changes in cell wall polysaccharide composition [19, 20, 25], modifies the pathogen's sensitivity to environmental stress and antifungals [18, 20, 23, 25], but also affect its immunogenicity by altering the expression and presentation of critical pathogen-associated molecular patterns (PAMPs), thus making *C. albicans* a moving target for the recognition by the host immune system [19, 20, 21•, 25, 26, 27].

The human host withholds the availability of micronutrients, like Fe, Zn, Cu and Mn, from the pathogen in a process termed ‘nutritional immunity’ [28]. These micronutrients are essential for many vital cellular functions in the pathogen [28]. Countering this, *C. albicans* has evolved mechanisms to overcome host nutritional immunity by expressing micronutrient transporters (e.g. Rbt5/Als3 for Fe; Zrt1/Zrt2/soluble Pra1 for Zn) [28], or redundant enzymes that use alternative micronutrients as cofactors [29].

*C. albicans* expresses a number of proteases, phospholipases, lipases and esterases (reviewed in [30]), which function in the degradation of host connective tissues, cleavage of host immune defence proteins, and thus aid nutrition acquisition, invasion and evasion of the pathogen from host immune defence [30, 31]. These hydrolases belong to multigene families, and each family member has a different substrate specificity, pH optimum, and expression profile [30]. Of these the secreted aspartyl proteases (Saps) have been shown to have important multiple roles. Regulation of the expression of this 10-membered gene family is regulated principally by host carbon and nitrogen sources and by pH [32]. Sap2 inactivates Factor-H and the complement receptors CR3 and CR4 on macrophages, thus mediating the escape of *C. albicans* from recognition by the host's innate immune system [33]. In mice, Anti-Sap2 antibodies or the protease inhibitor pepstatin A can reduce Sap2-mediated vaginal inflammation caused by *C. albicans* [34]. Sap2 and Sap6 have also been shown to induce inflammatory cytokine production by the host through the type I IFN, and caspase-11 induction as well as NLRP3 inflammasome activation [35, 36]. Following their uptake by the host epithelial cells, hypha-specific Saps4-6 cause lysosomal permeabilization and triggering of caspase-1 dependent apoptosis [37].

Recently, an entirely novel secretory host cell lysing agent from *C. albicans*, called ‘candidalysin’, has been described. The lytic activity is encoded by one of eight Kexp-processed peptides from the *ECE1* gene product. The Ece1-III^62–93^ peptide alone can induce damage to epithelial membranes and the activation of a danger response in host epithelial immunity [38^••^].

**Figure 1:**
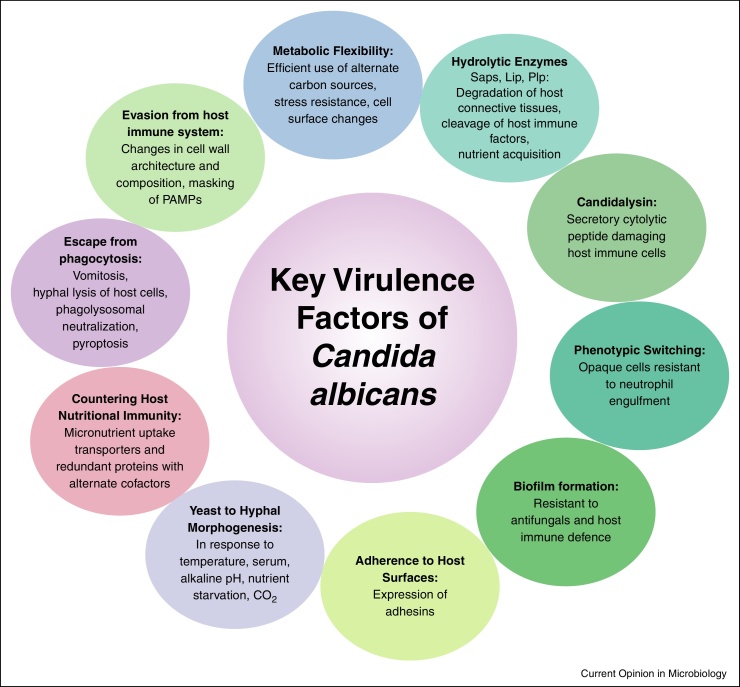
*Candida* virulence factors. Virulence is a polygenic trait in *C. albicans* involving biochemical, physiological, genetic and morphogenetic characteristics.

These evolutionary adaptive traits enable *C. albicans* to survive in various host niches, counter host immune defences and help *C. albicans* in establishing itself as a successful pathogen (Figure 1).

**Figure 2:**
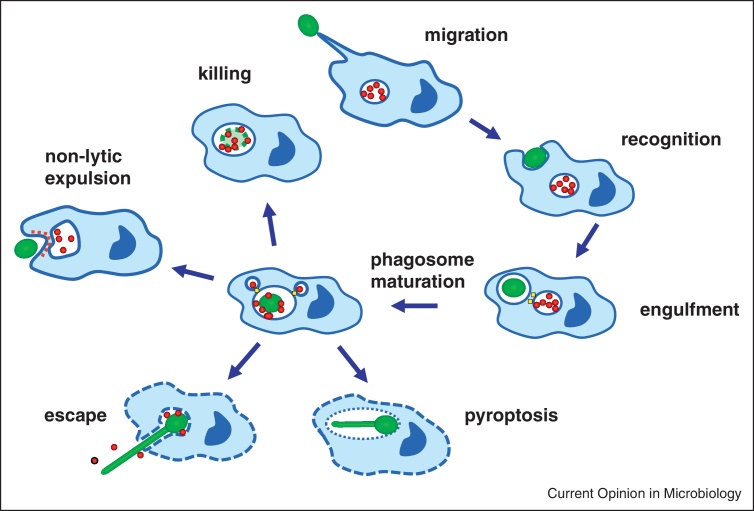
Interaction of *C. albicans* with macrophages showing the stages at which the fungus can deploy immune avoidance mechanisms. The macrophage uses chemotaxis to target the fungal cell, then fungus recognition and engulfment take place via interaction of fungal PAMPs and a series of immune cell PRRs that includes phagocytic receptors. The *Candida* cell is delivered to the phagosomal vesicle which undergoes fusion with lysosomes to create the mature acidic phagolysosome that can kill the fungal cargo using oxidative and nitrosative elements. However, *C. albicans* has the capacity to interfere with the phagolysomal maturation programme, increase its alkalinity, generate protective antioxidants and induce its own non-lytic expulsion. In addition, hypha formation and induced pyroptosis can cause the lysis of the phagocyte.

## Controlling interactions with human epithelia and endothelia

**Figure 3:**
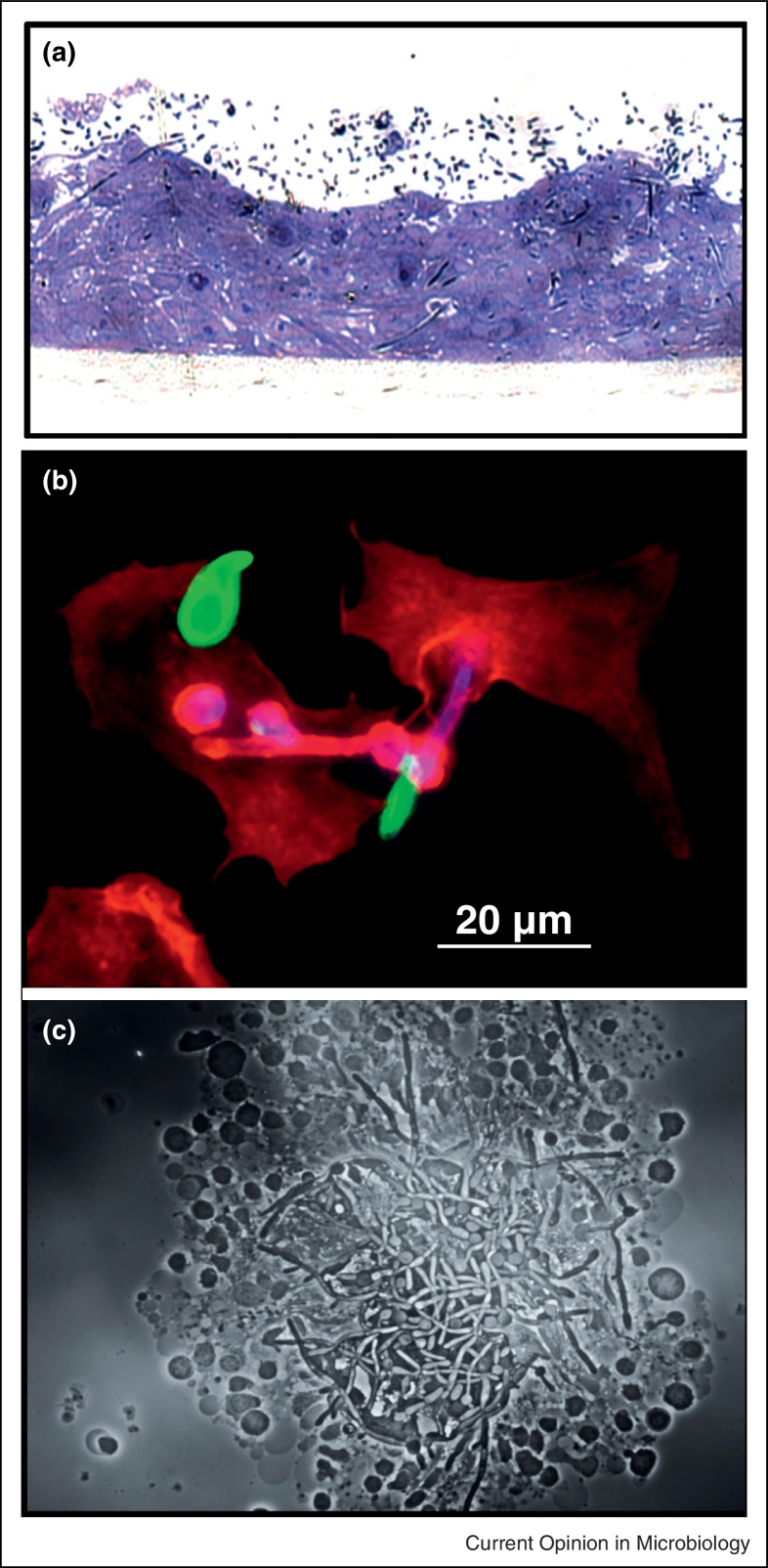
*C. albicans* interaction with host cells. **[a]** I invasion into oral epithelium with hyphae that have penetrated the host cells marked (*). **[b]** Phagocytosis by mouse peritoneal macrophages with extracellular parts of *C. albicans* stained with an anti-*Candida* Ab in green and counterstained with CFW in blue. The macrophages are stained red with phalloidin (F-actin) to visualize phagocytic uptake. **[c]** Human peripheral blood mononuclear cells aggregated around yeast cells and hyphae of *C. albicans*.

Two complementary mechanisms are involved in *C. albicans* host cell invasion through epithelial cells (EC) (Figure 3). Fungal-induced endocytosis contributes to the early stages of invasion, a process of host produced pseudopods surrounding the fungus to pull it into the host cell. By contrast, active penetration occurs at later time points when hyphae invade between or through ECs [39]. Although the mechanisms involved depend on invasion stage, fungal morphology and epithelial lineage, both are triggered by hypha-associated factors [40]. Notably, systemic dissemination, which is not dependent on morphological transition, occurs from the GI tract and trans-epithelial transport of the yeast form might be mediated by indirect mechanisms, such as by lumen sampling dendritic cells or M cell transcytosis [7].

Both the oral and vaginal epithelial immune response differs to the presence of hyphae and yeast cells of *C. albicans* so that only large numbers of hyphae activate a host biphasic MKP1 and c-Fos dependent response leading to inflammatory cytokine formation [41]. This may allow the epithelium to control the pattern of immune activation so it is responsive mainly to fungal invasion rather than colonisation. Low level colonisation, may progress to the establishment of a mature surface biofilm, but as yet it is not clear how the establishment of a *Candida* biofilm may affect mucosal infection and immunity [42]. However, it would seem likely that mature biofilms would present challenges for the cellular immune system to clear such dense biomass mats.

Multiple stimuli including EC contact and body temperature trigger hyphal morphogenesis. *C. albicans* produces early virulence factors that are downstream of the process of invasion itself [43]. These include the expression of the GPI-linked cell surface proteins Hwp1 and the invasin Als3 or Ssa1, a member of HSP70 family [40]. Hwp1, under control of the transcription factor Bcr1 regulating biofilm formation, acts as a substrate for epithelial transglutaminases and is required for mucosal pathogenicity [39]. Als3 and Ssa1 mediate binding to host epithelial surface receptors which enable the fungus to attach to and invade host cells. Host ligands include E-cadherin on ECs, N-cadherin on endothelial cells and EGF receptor on oral ECs [40]. During induced endocytosis this interaction stimulates host cytoplasmic proteins to form clathrin-coated vesicles surrounding the fungus for an actin dependent internalization. E-cadherin interaction is however not necessary for endocytosis of *C*. *albicans* into enterocytes [44]. Recently it has been shown that *C. albicans* Als adhesins are also involved in adhesion to *C. glabrata* during oropharyngeal candidiasis (OPC) enabling *C. glabrata* to colonize and persist in the oral mucosa and cavity [45^•^].

During active penetration, *C. albicans* secretes hydrolytic enzymes (described in previous section) that affect epithelial cell–cell junctions and facilitate degradation of cell membrane components along with other ligands that facilitate fungal adhesion [39]. Cell surface localized superoxide dismutase detoxifies reactive oxygen species (ROS) produced when tissue is damaged and is also expressed on the surface of invading hyphae [46, 47].

Many other internal and cell wall-associated proteins also indirectly affect *C. albicans* adhesion and virulence, for example, proteins involved in protein trafficking and required for a functional vesicle transport system or proteins involved in hyphal formation, growth or cellular orientation. Others have a crucial role in cell wall assembly and integrity, or they modify other adhesins required for epithelial binding [39].

## Interactions with phagocytes

Almost every component of the cell wall of *Candida* has been shown to have a role in the interactions between host and pathogen. Immune recognition of *Candida* is mediated by PAMP engagement with host pattern recognition receptors (PRRs). These recognition events are dominated by binding of fungal cell wall carbohydrates, although the cell wall proteins also contribute to shaping the immune response [3, 4]. Recently it has become clear that the innate immune response, which is of principle importance in conferring immunity, can be trained by prior exposure to *Candida*. As a consequence memory of prior innate immune interactions is epigenetically imprinted in the host and results in enhanced immune responses in subsequent encounters with the fungus. The paradigm of immunological memory was thought previously to be encoded entirely by the adaptive immune system but is clearly also a feature of innate immunity [48••, 49].

It is also increasingly evident that the immune response to *Candida* is influenced by yeast-hypha morphogenesis [50]. Hyphae specifically activate the NLRP3 inflammasome resulting in IL-1β secretion [51] and yeast and hyphae are differentially bound to, taken up and processed by phagocytes [3]. In addition, other morphological transitions, such as the switching from the white to the opaque form of this organism results in differential phagocytosis and the suppression of the production of phagocyte chemoattractants [52, 53].

## Resisting phagocytes

Following internalisation of the fungal target cell, maturation of the phagosome into phagolysosome is fundamental to fungal killing. The importance of phagolysosomes in promoting fungal killing is associated with the presence of cationic peptides, hydrolytic enzymes, ROS and reactive nitrogen species (RNS); which are generated in a vacuole with an acidic pH [3, 54]. Hence, in order to survive the harmful environment of the phagolysosome, *C. albicans* has developed strategies to allow its survival by the manipulation and escape from phagocytic cells (Figure 2, Figure 3) [54, 59].

*C. albicans* promotes neutralization of the phagolysosome, in a process that requires the Stp2 transcription factor induction of the Ato5-mediated ammonia exporter [55, 56•]. The subsequent neutralization of the acidic environment of the phagolysosome allows *C. albicans* hyphal morphogenesis to occur hence facilitating its escape from macrophages [55, 56•]. In addition, the PKC-Mkc1-Rlm1 cell wall salvage pathway is also activated resulting in reinforcing chitin being made in the cell wall [57, 58].

When inside the phagosome *Candida* also induces a battery of protective enzymes and proteins including catalase, superoxide dismutase, thioredoxin, flavo-haemoglobin, glutathione recycling enzymes that degrade or scavenge RNS and ROS [47, 60]. Consequently, deletion of key regulators of oxidative and nitrosative stress response results in increased sensitivity to phagocyte killing [47, 60]. Multiple individual imposed toxic stresses have been shown to exert synergistic effects on the ability to kill *Candida*. These studies illustrate the importance of the Hog1 MAPK and the Cap1 transcription factor in the regulation of combinatorial stress responses [60, 61].

*Candida* cells share a property with *Cryptococcus* and other fungi to be able to induce their own non-lytic expulsion from macrophages (sometimes called vomocytosis), without any damage to the phagocyte [62]. By contrast, numerous studies have reported the ability of elongating hyphae to pierce the membrane of a macrophage resulting in their release [3]. However, there is no clear relation between yeast-hypha morphogenesis and macrophage lysis.

It is now clear that *Candida* hyphal cells can also induce pyroptosis in the host phagocyte — a form of programmed cell death that is dependent on caspase-1 activation of the NLRP3 inflammasome [63, 64, 65, 66]. However, although hyphae promote pyroptosis, pyroptosis is not always coincident with hypha formation. For example, a *vps35* mutant formed hyphae normally but failed to induce macrophage lysis whilst, *alg1* and *alg11* mutants are hypha-deficient yet induced macrophage lysis via pyroptosis [67^••^].

Therefore the cell biology of *Candida* is well adapted to resist the killing potential of phagocytes thereby limiting the effectiveness of some elements of the adaptive immune system.

## Conclusion

*C. albicans* has numerous cell biology attributes that enable it to exist commensally in the human body. These features presumably account for the fact that most healthy humans are colonised by this organism. However, its metabolic, morphogenetic and immunomodulatory properties means that it is also a pernicious and common pathogen in almost any setting in which immune vigour is compromised or the physical integrity of the host surface is disrupted. As a commensal organism *Candida* has likely acquired traits and properties that also enable it to flourish as a pathogen [68]. Commensal cells also have to avoid being eliminated by overcoming immune surveillance mechanisms that act to protect the mucosa. This organism can therefore ‘have its cake and eat it’ by withholding its full pathogenic potential under conditions of host immune fitness or aggressively invading the weakened host. In both situations this fungus shows remarkable flexibility and adaptability in its capacity for survival in the human body.

## References and recommended reading

Papers of particular interest, published within the period of review, have been highlighted as:

• of special interest
•• of outstanding interest
